# Use and Utility of Web-Based Residency Program Information: A Survey of Residency Applicants

**DOI:** 10.2196/jmir.5.3.e22

**Published:** 2003-09-25

**Authors:** Peter J Embi, Sima Desai, Thomas G Cooney

**Affiliations:** ^1^Oregon Health & Science UniversityDepartment of MedicinePortland ORUSA

**Keywords:** Internship and residency, Internet, education, medical, graduate, job application, career choice, pamphlets

## Abstract

**Background:**

The Internet has become essential to the residency application process. In recent years, applicants and residency programs have used the Internet-based tools of the National Residency Matching Program (NRMP, the Match) and the Electronic Residency Application Service (ERAS) to process and manage application and Match information. In addition, many residency programs have moved their recruitment information from printed brochures to Web sites. Despite this change, little is known about how applicants use residency program Web sites and what constitutes optimal residency Web site content, information that is critical to developing and maintaining such sites.

**Objective:**

To study the use and perceived utility of Web-based residency program information by surveying applicants to an internal medicine program.

**Methods:**

Our sample population was the applicants to the Oregon Health & Science University Internal Medicine Residency Program who were invited for an interview. We solicited participation using the group e-mail feature available through the Electronic Residency Application Service Post-Office application. To minimize the possibility for biased responses, the study was confined to the period between submission of National Residency Matching Program rank-order lists and release of Match results. Applicants could respond using an anonymous Web-based form or by reply to the e-mail solicitation. We tabulated responses, calculated percentages for each, and performed a qualitative analysis of comments.

**Results:**

Of the 431 potential participants, 218 responded (51%) during the study period. Ninety-nine percent reported comfort browsing the Web; 52% accessed the Web primarily from home. Sixty-nine percent learned about residency Web sites primarily from residency-specific directories while 19% relied on general directories. Eighty percent found these sites helpful when deciding where to apply, 69% when deciding where to interview, and 36% when deciding how to rank order programs for the Match. Forty-nine percent found sites most useful in deciding where to apply, while 40% found them most useful while preparing for their interviews. Seventy-two percent felt that a "complete" Web site could substitute for a mailed printed brochure. Qualitative analysis identified additional important information needs.

**Conclusions:**

Applicants are turning to residency Web sites for information during critical phases of the application process. Though usually helpful, many of these sites are felt to be incomplete and may not be meeting important applicant information needs. These findings should be useful to those involved in residency recruitment efforts and in counseling applicants.

## Introduction

The Internet has dramatically changed the residency application process. The process, which used to rely exclusively on the exchange of printed materials between applicants and residency programs, has become largely Web-based.

Starting in 1995, applicants participating in the National Residency Matching Program (NRMP) have used the Electronic Residency Application Service (ERAS) to complete and submit their residency applications, initially via Dean's Office Workstations and currently via a Web-based system (MyERAS *)*. Similarly, applicants and programs submit and receive their Match information via the NRMP's Web-based system. In addition to its growing role in the management of the application and matching process, the Web is changing another significant aspect of the application process: the distribution of residency program information.

Little is known about how applicants currently obtain and use residency program information. In the pre-Web era, the principle sources of such information were printed brochures and the AMA-FREIDA (American Medical Association-Fellowship and Residency Electronic Interactive Database Access) database [1]. With the advent of the Web, residency programs began to place their program information on Web sites, and many ceased to provide printed materials [2,3]. Despite this, a recent MEDLINE/PubMed search identified only one other study that evaluated the usefulness of these sites to applicants [4]. To better understand how applicants use residency Web sites and what information would be most helpful to them as they progress through the application process, we studied the use and utility of Web-based residency program information by surveying applicants to an internal medicine program.

## Methods

Our sample population consisted of the 431 applicants to the Oregon Health & Science University Internal Medicine Residency Program who were invited for an interview. To minimize the potential for bias due to participants' perception that their responses might influence their NRMP rank by the residency program, we conducted the survey during an 18-day interval between the deadline for submission of NRMP rank-order lists and the date that the NRMP results were released.

We developed a survey containing a series of multiple-choice and free-text-entry questions and conducted it via the Internet. There were two reasons for conducting the survey using an Internet-based method. First, we knew that all subjects were e-mail and Web users as this was a requirement of engaging in the NRMP application process. Second, by conducting the survey via the Internet, we assured data collection precisely during the defined narrow window of time referred to above, a feat that would have been impossible using a traditional mail survey. Considering the preferences or limitations of Internet-based survey participants, we provided the option of responding to the survey via e-mail or via the Web in the hope of maximizing responses [5].

The Web-based survey was authored as a simple HTML form and was processed using a CGI (Common Gateway Interface) script (FormMail V1.9 copyright 1995-2001 Matt Wright). The form allowed for one response to each multiple-choice question and unlimited free-text entry for the comment questions. Upon submission of the Web-based survey, responses were immediately transmitted to the study's principle investigator as an anonymous e-mail message. The e-mail was identified as relating to this study in the subject line and included the date, time, and response information, but no respondent identifying information. If respondents opted to reply via e-mail instead of via the Web-based survey, their answers were extracted from the reply and transferred to another file, eliminating any identifying information.

Before deploying the survey, we solicited feedback from current residents at our program. We also tested the Web page's display characteristics and functionality using various computer operating systems (Microsoft Windows 95, 98, NT; and Mac OS 8.6, 9.0), Web browsers (Microsoft Internet Explorer 4.0, Netscape Communicator 4.5), and types of Internet access (modem dial-up, cable-modem broadband, high-speed local area network). The pilot tests did not uncover any technical problems, and reviewers reported that the survey and its instructions were clear and easy to use.

As is the case with all applicants to accredited US internal medicine residency programs, our sample population used the ERAS system throughout the NRMP application process. We used the group e-mail feature in the ERAS Post Office system to send the selected applicants an e-mail message. The message included a brief explanation of the survey's purpose, a request to take part in the survey, an assurance of anonymity, instructions describing the two ways participants could respond, and the survey itself. Respondents could either follow the included hyperlink to a Web-based version of the survey or they could reply to the e-mail message with their answers typed alongside the survey questions.

The initial e-mail message was sent on March 5, 2001, and two follow-up messages were sent to all subjects during the study period. On March 22, 2001, the day residency match results were released, we removed the survey from the Web site and ignored any subsequent e-mail replies received.

Survey responses were transferred to a spreadsheet (Microsoft Excel) for tabulation and we calculated percentages for each response based on the total number of responses to each question. Two of the study's investigators performed a qualitative analysis of the free text comments, assigning each comment to a category. A third reviewer resolved any discrepancies.

## Results

Table 1 describes the characteristics of those invited to interview and participate in the study compared to the national cohort of applicants who applied through ERAS to internal medicine residency programs (Teresa Bay, AAMC-Association of American Medical Colleges-personal communication, 2001). Of the 431 potential subjects contacted, 218 responded to the survey (51%). Eighty-nine percent of our participants responded through the Web-based survey while the other 11% responded directly by e-mail. The majority of applicants to our institution were US citizens, mostly from US schools, evenly split between males and females. Thirty-eight percent of our invited applicants were from western states (165/431). On the national level, applicants to internal medicine residency programs included more international applicants (54%), a male to female predominance (59% vs 41%) and a larger percentage of applicants from the US Northeast.

**Table 1:** Internal medicine applicant demographics

	**Study** **Population**	**National ERAS** **Population**
Gender Male Female	216 (50.1%) 215 (49.9%)	9353 (59.3%) 6422 (40.7%)
Citizenship US citizen Foreign national Permanent resident Conditional permanent	412 (95.6%) 7 (1.6%) 11 (2.6%) 1 (0.2%)	9040 (57.1%) 3442 (21.7%) 3075 (19.4%) 275 (1.7%)
Medical school type US public US private Canadian Osteopathic Fifth Pathway International	271 (62.9%) 151 (35.0%) 2 (0.5%) 3 (0.7%) 0 (0.0%) 4 (0.9%)	3536 (22.3%) 3004 (19.0%) 47 (0.3%) 605 (3.8%) 58 (0.4%) 8582 (54.2%)
Home regions West Midwest South Northeast Quebec None listed	165 (38.3%) 98 (22.7%) 85 (19.7%) 72 (16.7%) 3 (0.7%) 8 (0.2%)	662 (4.2%) 1505 (9.5%) 3388 (21.5%) 5022 (31.8%) 41 (0.3%)

Survey responses are summarized in Table 2. There were no notable differences in the responses of those replying via the Web versus those replying via e-mail. Most respondents were very comfortable browsing the Web (85.6%). The majority of respondents (78.1%) reported conducting at least some of their Web browsing from home while a substantial minority (20.9%) accessed the Web primarily from school/hospital. Of the applicants, 68.7% learned about residency program Web sites from residency specific directories like those found on organizational Web sites or AMA-FREIDA, while 18.9% discovered them using general Web directories. Only 4.1% of the participants learned of residency Web sites directly from the residency programs and 6.4% from colleagues or resources at their schools.

A majority of respondents found the Web sites helpful in deciding where to apply (79.6%) and where to interview (68.5%), and a substantial minority (35.8%) found them useful when rank-ordering programs for the NRMP Match. Web sites were most helpful in deciding where to apply (48.8%) and in preparing for the visit/interview (39.6%). About half of the respondents found a mailed program brochure unnecessary if the program had what was described simply as a "complete" Web site. An additional 21.3% indicated no need for a mailed brochure if the Web site provided a printable version of their program information. Of the respondents, 28.3% considered most (76%-100%) of the residency programs to have a "complete" Web site, while 25.4% reported that 50% or fewer Web sites were "complete."

Table 3 summarizes the information applicants would like to see added to residency Web sites, based on a qualitative analysis of their comments. Major information needs included: schedule information, career/fellowship placement, resident information, benefits information, contact information, and city information.

Comments included this typical quote from a respondent who reported wanting, "just all the details residency schedules, vacation times, information about their interview and ranking process. The nuts-and-bolts. It's frustrating when you can find some but not all of those basic details which are scattered on 15 pages." Another wrote, "more information regarding typical intern schedules, policy on admission caps, research, bench and clinical."

**Table 2:** Residency Web site survey responses

**Survey Questions**	**Response**	**Percentages**
1. How comfortable are you at browsing the World Wide Web?	Very Somewhat Uncomfortable	85.6% 13.4% 0.9%
2. From where do you usually access the Web?	Home School/Hospital Equal home/school Other	52.1% 20.9% 26.0% 0.9%
"3. How did you most commonly learn about residency programs" Web sites?	Colleagues/School resources General Web directory/search engines Residency/Medicine directories Information from residency programs Other	5.9% 18.9% 68.7% 4.1% 2.3%
4. Did you find the residency program Web sites helpful when deciding where to apply?	Yes No	79.6% 20.4%
5. Did you find the residency program Web sites helpful when deciding where to interview?	Yes No	68.5% 31.5%
6. Did you find the residency program Web sites helpful when deciding how to rank-order programs in the "Match"?	Yes No	35.8% 64.1%
7. At what point in the application process did you find program Web sites most useful?	""Deciding where to apply Deciding where to interview Preparing for visit/interview Deciding rank-order for the "Match" Other	48.8% 6.9% 39.6% 3.2% 1.4%
8. If a program has a "complete" Web site, do you feel that an additional printed brochure is necessary?	Printing from web site adequate Mailed printed brochure Complete web site sufficient	21.3% 28.2% 50.5%
9. Of the residency programs to which you applied, how many had "complete" Web sites?	1%-25% of residency Web sites 26%-50% of residency Web sites 51%-75% of residency Web sites 76%-100% of residency Web sites	4.2% 21.2% 46.2% 28.3%

**Table 3:** Qualitative analysis of respondent comments to the question: "What kind of information would you like to see added to residency program Web sites, in general?"

**Comments**	**Number of Responses**
1. Schedule information/schedule access	34
2. Career/fellowship placement	19
3. Resident information (medical school, biographical, etc)	19
4. Residency benefits information	11
5. Residency contact information (program, residents, faculty, interviewer)	11
6. City information (general info, housing, cost of living)	11
7. Research information	6
8. Residency elective information	6
9. Program vision/goals (philosophy)	4
10. Unique program features	4
11. Testimonials (resident, faculty)	4
12. Board pass rate	3
13. Differentiation primary care/categorical	3
14. Rotation/medical service details	3
15. Hospital information	3
16. Faculty profiles	3
17. Frequently asked questions/answers	3
18. Photos of facility/personnel	2
19. Area jobs information	2
20. Printable program information	2
21. Detailed map of campus/directions	2
22. Conference information	2
23. Workload/cap information	2
24. Optimizing Web site design/organization	2
25. Access to actual program information resources	1
26. Detailed application information	1
27. Details of interview/ranking process	1
28. FREIDA-like information	1

## Discussion

Residency applicants and programs are increasingly using the World Wide Web for information gathering and dissemination during the residency application process. Until now, there has been little data available in the published literature to inform those developing residency program Web sites about the needs and usage patterns of prospective residency applicants. These findings offer some insight into how applicants use these sites and what they expect from them, information that should be useful to those engaged in applicant counseling and recruitment efforts.

As part of the application process, all applicants must use the Internet, so it is not surprising that most respondents to our survey were very comfortable browsing the Web. Our finding that most applicants primarily browse the Web from home should be taken into consideration by residency programs as they design content for their Web sites. While applicants' home connection speeds will likely improve as more households adopt faster broadband Internet connections, most are likely still accessing the Web via slower dial-up modem connections and may therefore be limited in the size of data files that can be efficiently downloaded and viewed [6].

These findings also provide insight into how applicants learn about program Web sites. While most relied on residency and specialty-specific directories, a significant minority used general Web directories and search engines. This suggests that residency programs can maximize the likelihood that prospective applicants will discover their Web site by listing and keeping updated links to their sites on such Web-based directories and search engines.

Once applicants reach residency Web sites, they use the sites to varying degrees during virtually every stage in the application process, from initial consideration of programs to creation of rank-order lists. Respondents found the sites most useful when deciding where to apply and when preparing for program visits; considering what information is pertinent to those aspects of the application process may help programs determine the Web site content to enhance.

As residency programs move toward displaying their information on Web sites, many are abandoning their printed brochures for Web-only offerings [7]. This can certainly yield benefits, including cost-savings and timelier updating of content, but the consequences of moving away from traditional methods of disseminating information to prospective applicants are not fully known [2]. While our finding that most respondents felt a "complete" Web site or the ability to print program information obviated the need for a printed brochure, 28.2% still wanted to receive a printed brochure by mail. This appears to be an improvement over the 50% level noted in the other published survey of a similar population, which was conducted during the 1997-1998 interview season, a finding that may indicate that the preference for printed brochures is declining over time [4]. Nevertheless, some programs may wish to consider these findings as they contemplate whether to abandon printed brochures.

While the meaning of Web site "completeness" remains ill defined, fewer than one third of respondents perceived most (76%-100%) residency Websites to be "complete." This reinforces the observation noted by other researchers that residency Web sites vary widely in their content and thus usefulness, and suggests that Web site content managers should consider enhancing their online residency information offerings [7].

Providing optimally-useful information on residency Web sites requires an understanding of applicants' information needs. Our qualitative analysis helps illuminate what applicants perceive to be their current unmet information needs on such sites. Their comments focused on a range of academic, financial, career, and personal information, further reinforcing the contention that, while certainly helpful, residency Web sites on the whole still have room to improve in meeting applicants' information needs.

As noted above, our literature review identified only one other study that attempted to assess how residency applicants access or utilize information at any stage of the application process [4]. The current study's findings improve our understanding of this area, but this is clearly an area in need of further research given the remaining unanswered questions, the dynamic nature of the Web, and the impacts that such shifts in information exchange can have on a process as important as residency selection and recruitment.

Our study has limitations. First, it was limited to the invited applicants of one specialty program. Our population differed from the national cohort in that there were far fewer international graduates and a greater percentage of our invited applicants were from the western United States. Second, because we elected to use an anonymous response strategy, we cannot determine if responders differed from nonresponders. Third, because we used e-mail and a Web-based survey, it is possible that we selected for a population more favorably inclined toward use of electronic resources.

### Conclusions

Residency applicants and programs increasingly rely on the Web to gather and receive information during the application process. Little data has been available in the published literature to inform those managing residency Web sites about the needs and usage patterns of applicants. While further study in this area is needed, these findings provide much needed insight into how applicants use these sites and what they expect from them, information that should be considered by those engaged in residency promotion and recruitment efforts.

## Abbreviations

AMA: American Medical Association
ERAS: Electronic Residency Application Service
FREIDA: Fellowship and Residency Electronic Interactive Database Access
NRMP: National Residency Matching Program
